# Adverse Outcomes Associated With Corticosteroid Use in Critical COVID-19: A Retrospective Multicenter Cohort Study

**DOI:** 10.3389/fmed.2021.604263

**Published:** 2021-02-10

**Authors:** Yichen Li, Jie Li, Jia Ke, Na Jiao, Lixin Zhu, Lihan Shen, Lei Chen, Zhiqiang Jiang, Sijing Cheng, Yibo Huang, Yifeng Zou, Ruixin Zhu, Guangjun Yan

**Affiliations:** ^1^The Sixth Affiliated Hospital, Sun Yat-sen University, Guangzhou, China; ^2^The Third Clinical Medical College of Yangtze University, Jingzhou Hospital of Traditional Chinese Medicine, Jingzhou, China; ^3^Putuo People's Hospital, Department of Bioinformatics, Tongji University, Shanghai, China; ^4^Department of Critical Care Medicine, Dongguan People's Hospital, Southern Medical University, Dongguan, China; ^5^Department of Critical Care Medicine, The Sixth Affiliated Hospital, Sun Yat-sen University, Guangzhou, China; ^6^School of Medicine, Sun Yat-sen University, Shenzhen, China

**Keywords:** SARS-CoV-2, mortality, critical, gluocorticoid, steroid, COVID-19, ARDS, cytokine storm

## Abstract

Corticosteroid is commonly used to reduce damage from inflammatory reactions in coronavirus disease 2019 (COVID-19). We aim to determine the outcomes of corticosteroid use in critically ill COVID-19 patients. Ninety six critically ill patients, hospitalized in 14 hospitals outside Wuhan from January 16 to March 30, 2020 were enrolled in this study. Among 96 critical patients, 68 were treated with corticosteroid (CS group), while 28 were not treated with corticosteroids (non-CS group). Multivariable logistic regression were performed to determine the possible correlation between corticosteroid use and the treatment outcomes. Forty-six (68%) patients in the CS group died compared to six (21%) of the non-CS group. Corticosteroid use was also associated with the development of ARDS, exacerbation of pulmonary fibrosis, longer hospital stay and virus clearance time. On admission, no difference in laboratory findings between the CS and the non-CS group was observed. After corticosteroid treatment, patients treated with corticosteroids were associated with higher counts of white blood cells, neutrophils, neutrophil-to-lymphocyte ratio, alanine aminotransferase level and Sequential Organ Failure Assessment score. In conclusion, corticosteroid use in critically ill COVID-19 patients was associated with a much higher case fatality rate. Frequent incidence of liver injury and multi-organ failure in corticosteroid treated patients may have contributed to the adverse outcomes. The multi-organ failure is likely caused by more persistent SARS-CoV-2 infection and higher viral load, due to the inhibition of immune surveillance by corticosteroid.

## Introduction

The pandemic of Coronavirus Disease 2019 (COVID-19) has caused many deaths, and no therapy with proven efficacy is available. The viral pathogen of COVID-19 is severe acute respiratory syndrome-related coronavirus-2 (SARS-CoV-2), which, together with SARS-CoV, belongs to the species of SARSr-CoV (Severe acute respiratory syndrome-related coronavirus) (1). Both viruses are closely related to MERS-CoV, which causes Middle East respiratory syndrome (MERS).

Study with SARS patients indicated that immune-mediated mechanism rather than virus induced damage drives the clinical progression (2). Consistently, study with macaques indicated that severe symptoms of SARS infection were caused by elevated immune reactions rather than higher viral load, and the anti-inflammatory therapy with type I interferon reduced the respiratory symptoms (3). Similar to SARS and MERS (4), high levels of proinflammatory cytokines and chemokines, the cytokine storm, were observed in the peripheral blood of COVID-19 patients (5). Based on the pathological findings including significantly increased serum level of cytokines, and over-activation of T cells in severe COVID-19 patients, Xu et al. recommended to treat COVID-19 with corticosteroid to control immune reactions (6). Corticosteroid has been commonly administered to COVID-19 patients in (7, 8) and outside China (9).

However, the application of corticosteroids in coronavirus infection has consequences. The use of corticosteroids in SARS patients was associated with serious complications including avascular necrosis, diabetes and psychosis (10). Corticosteroid use also led to delayed viral RNA clearance in SARS (11) and MERS (12). In addition, suppression of the patients' immune system with corticosteroids leads to secondary infection, as observed in clinical trials of septic shock (13) and respiratory failure (14). To understand the beneficial and adverse effects of corticosteroid use in COVID-19, we reviewed the medical records of critical COVID-19 patients from 14 hospitals and found that the use of corticosteroid is associated with more severe respiratory symptoms and a much higher case fatality rate.

## Methods

### Study Design and Participants

**Figure 1:**
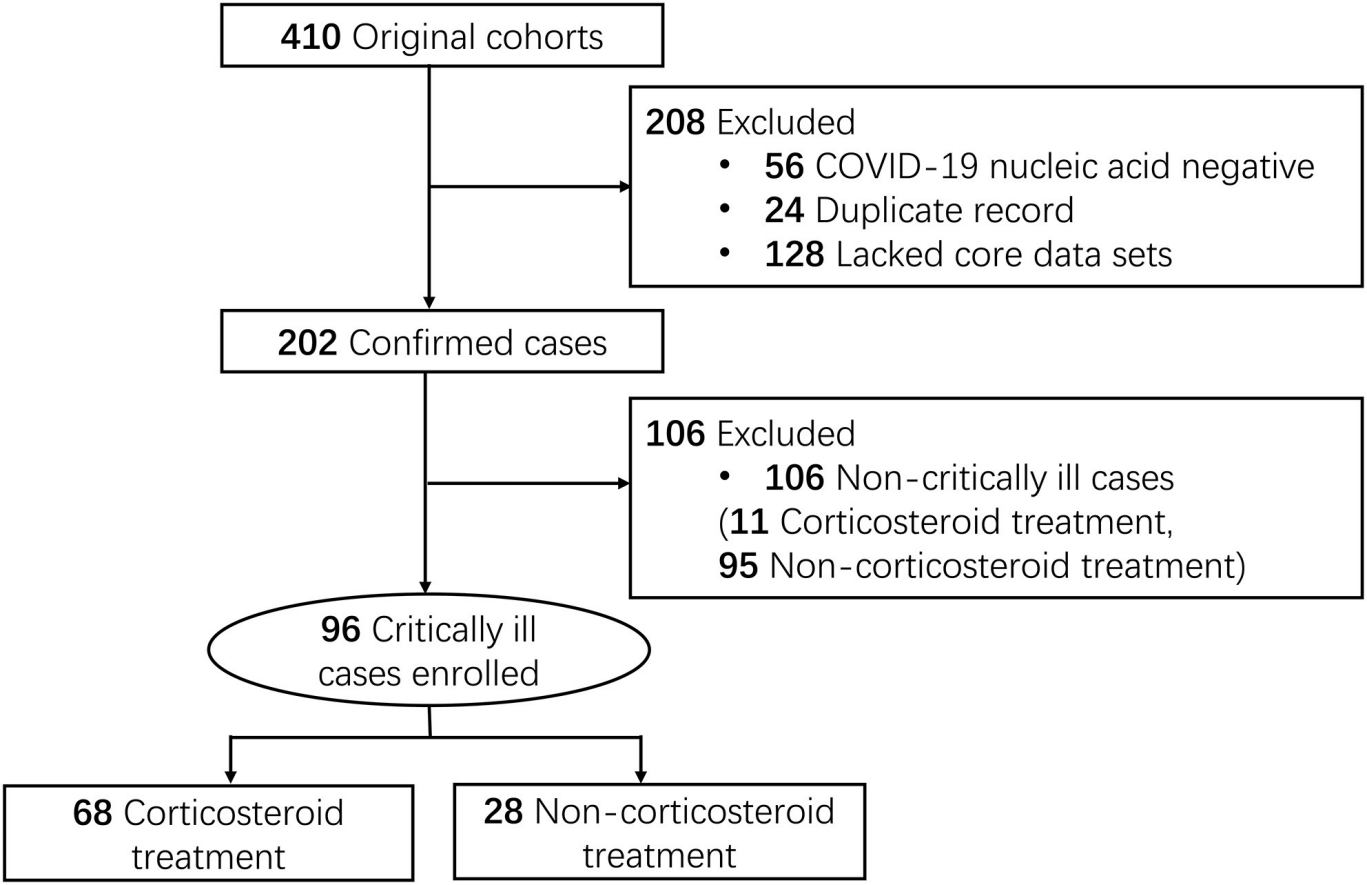
Study flow diagram. Medical records of COVID-19 patients (106 non-critically ill and 96 critically ill) included for the study of the corticosteroid effects were accessed from Jingzhou Hospital of Traditional Chinese Medicine (61 cases), Jianli Hospital of Traditional Chinese Medicine (44), Jingzhou First Hospital (9 cases), Jingzhou Central Hospital (29 cases), Jingzhou First People's Hospital (11 cases), Jingzhou Chest Hospital (9 cases), Gongan People's Hospital (8 cases), Honghu People's Hospital (10 cases), Honghu Hospital for the Control of Schistosomiasis (3 cases), Honghu Central Hospital (2 cases), Jianli People's Hospital (3 cases), Jiangling People's Hospital (2 cases), Shishou Hospital of Traditional Chinese Medicine (5 cases), and Songzi People's Hospital (6 cases).

We reviewed 410 patient charts of suspected COVID-19. Excluding 56 records for negative SARS-CoV-2 nucleic acid test, 24 for duplicated records, and 128 for the lack of relevant data, we identified 202 SARS-CoV-2 RNA positive patients hospitalized at 14 hospitals (Figure 1) in Jingzhou, China, from January 16 to March 30, 2020. Our COVID-19 patients were admitted to the hospitals because of fever, cough, dyspnea and chest computed tomography (CT) findings indicating SARS-CoV-2 pneumonia. Diagnosis of COVID-19 was based on positive SARS-CoV-2 nucleic acid test. Ninety-six critically ill patients were enrolled in this study. Critically ill patients were defined as those admitted to the ICU, requiring mechanical ventilation, or had a fraction of inspired oxygen (FiO2) of at least 60% (15, 16).

Treatment of the infection followed the fifth edition of the Diagnosis and Treatment Guideline for COVID-19, National Health Commission of the People's Republic of China. Besides oxygen supplementation and respiratory support, patients were routinely given antibiotics, usually Moxifloxacin, and antivirus drugs, usually Lopinavir and Ritonavir. Mechanical ventilation was conducted when hypoxemia and dyspnea persisted despite non-invasive oxygen supplementation.

The use of corticosteroid in COVID-19 patients is controversial, and this is reflected in the fifth edition of the Diagnosis and Treatment Guideline for COVID-19, National Health Commission of the People's Republic of China, which recommended optional use of corticosteroid when evidence presented for deteriorating (or persistent) dyspnea or chest CT results. Corticosteroid [at a dose not to exceed the equivalent of 1~2 mg methylprednisolone /kg/day, and should be used for a short period of time (3 to 5 days)] was prescribed according to physicians' preference. Sixty-eight of the critically ill patients were treated with corticosteroids. We compared the demographics, symptoms, treatments and outcomes between critical COVID-19 patients treated with corticosteroids (CS group) and those not treated with corticosteroids (non-CS group).

This study was approved by the Institutional Review Boards of Sun Yat-sen University, and participating hospitals. Informed consent was waived for this retrospective chart review.

### Data Collection

Charts were reviewed for demographic, clinical, laboratory, treatment and outcome data. Demographic data included age, gender, and comorbidities including hypertension, diabetes, cardiovascular disease, chronic obstructive pulmonary disease, chronic liver disease and malignancy. Clinical data recorded included vital signs such as temperature, respiratory rate, blood pressure, and oxygen saturation. In addition to fever, cough and dyspnea, other clinical characteristics recorded included sputum production, diarrhea (three or more loose or liquid stools per day), bloody stool (stool positive for occult blood test or white blood cell test), myalgia/muscle fatigue and haemoptysis. Laboratory data included blood cell counts (white blood cell, lymphocyte, neutrophil, monocyte, and platelet), markers for coagulation function (activated partial thromboplastin time (APTT), d-dimer and fibrinogen), infection-related biomarkers (C-reactive protein (CRP), Erythrocyte sedimentation rate (ESR), neutrophil-to-lymphocyte ratio (NLR) and procalcitonin), and other blood biochemistry measurements (aspartate transaminase (AST), alanine aminotransferase (ALT), creatine kinase (CK), creatinine, blood urea nitrogen (BUN), lactate dehydrogenase (LDH), total bilirubin and Sequential Organ Failure Assessment (SOFA) score). Cytokines were not measured for most patients.

The hospital course was reviewed for treatments and severity of disease. The need for supplemental oxygen, respiratory support and admission to the intensive care unit (ICU) were recorded.

### Statistics

SPSS (Statistical Package for the Social Sciences) version 22.0 software (SPSS Inc.) was used for Student *t* test, Mann-Whitney *U* test, Chi-Square test, Fisher's exact test, multivariable Cox regression analysis, univariable and multivariable logistic regressions. Significance of the differences between two study groups were tested by Student *t* test or Mann-Whitney *U* test for numerical data, when appropriate. For multiple comparisons, *p*-values were adjusted by FDR method in R. Comparisons of categorical data were performed by Chi-Square test or Fisher's exact test, as appropriate. Age was transformed to categorical variable at the threshold of 60. Laboratory findings before discharge were transformed to categorical variables based on reference values. Multivariable logistic regressions were performed to identify clinical features and treatments associated with case fatality and corticosteroid treatment, adjusted for multiple potential confounders identified from univariable regressions. All statistical tests were two sided, with *p*-values of < 0.05 considered to be statistically significant.

## Results

### Demographics, Clinical Characteristics and the Treatments of the Critical COVID-19 Patients

A large proportion (68 of 96, 71%) of the critically ill COVID-19 patients were treated with corticosteroids, compared to 10% (11 of 106) of the non-critical patients (Figure 1). Therefore, this study only concerned the critical patients, among which 68 patients were treated with corticosteroids (CS group) while 28 patients were not treated with corticosteroid (non-CS group). Fifty-three patients (78%) in the CS group were 60 or older compared to 14 (50%) in the non-CS group (Table 1). No significant difference in gender ratio was found between the two groups.

**Table 1:** Demographics and clinical characteristics of critical COVID-19 patients on admission.

	**Corticosteroid treatment (*n* = 68)**	**Non-corticosteroid treatment (*n* = 28)**	***P*-value**
**Age, years**			
≥60	53 (78%)	14 (50%)	0.063
**Sex**			
Female	30 (44%)	10 (36%)	1.000
Male	38 (56%)	18 (64%)	
**Signs and symptoms**			
Fever (≥37.3°C)	59 (87%)	21 (75%)	0.594
Cough	50 (74%)	21 (75%)	1.000
Dyspnea	31 (46%)	12 (43%)	1.000
Sputum	22 (32%)	6 (21%)	0.852
Myalgia	18 (26%)	2 (7%)	0.439
Diarrhea	12 (18%)	3 (11%)	1.000
Cephalalgia	10 (15%)	2 (7%)	1.000
Hemoptysis	1 (1%)	1 (4%)	1.000
**Comorbidities**			
Hypertension	32 (47%)	12 (43%)	1.000
Diabetes	11 (16%)	5 (18%)	1.000
Digestive tract disease	6 (9%)	3 (11%)	1.000
Cardiovascular disease	7 (10%)	2 (7%)	1.000
Cerebrovascular disease	4 (6%)	1 (4%)	1.000
Malignancy	2 (4%)	1 (4%)	1.000
Liver disease	3 (4%)	1 (4%)	1.000
Chronic lung disease	6 (9%)	0	0.594
**Treatments**			
Antiviral treatment	61 (90%)	25 (89%)	1.000
Interferon	17 (25%)	0	**0.027**
Antibiotics	59 (87%)	26 (93%)	1.000
Immunoglobulin	20 (29%)	0	**0.027**
Albumin	5 (7%)	0	0.856
Thymosin α1	7 (10%)	2 (7%)	1.000
Supplemental oxygen	68 (100%)	26 (93%)	0.448
Mechanical ventilation	36 (53%)	10 (36%)	0.563
ECMO	1 (1%)	2 (7%)	1.000

*Data are n (%). P-values comparing corticosteroid treatment and non-corticosteroid treatment are from Chi-Square test or Fisher's exact test, as appropriate, and adjusted for multiple comparisons by FDR method. COVID-19, coronavirus Disease 2019; ECMO, extracorporeal membrane oxygenation. The bold P-values indicate P < 0.05*.

On admission, the most common symptoms for both groups were fever (87% in the CS group), cough (74%, CS), and dyspnea (46%, CS), while less common symptoms included sputum production (32%, CS), myalgia (26%, CS), diarrhea (18%, CS), cephalalgia (15%, CS), and hemoptysis (1%, CS). No difference in the incidences of these symptoms was observed between the CS group and the non-CS group (Table 1).

The most frequently recorded comorbidities for both groups of patients included hypertension (47%, CS), diabetes (16%, CS), and cardiovascular disease (10%, CS). No difference in the incidence of any comorbidity between the CS group and the non-CS group was observed (Table 1).

Following the diagnosis and treatment guideline of the National Health Commission (China), the most common treatments included supplemental oxygen (100%, CS), antiviral treatments (90%, CS), and antibiotics (87%, CS). No difference was observed for these treatments between the CS and the non-CS groups (Table 1). The two groups were also similarly treated with thymosin α1, mechanical ventilation and ECMO. It is noteworthy that some patients in the CS group were treated with interferon (25%), immunoglobulin (29%) and albumin (7%), which were not used for patients in the non-CS group (Table 1).

### Outcomes of the Patients Treated With or Without Corticosteroids

Forty-six of the 68 (68%) patients in the CS group died compared to six of the 28 (21%) in the non-CS group (Table 2). After adjusting for potential confounding factors including age, gender, hypertension, interferon, immunoglobulin, and thymosin α1, corticosteroids treatment was identified as a risk factor for case fatality in critically ill COVID-19 with an adjusted OR of 4.05 (95%CI: 1.37–11.98, *p* = 0.012, *R*^2^ = 0.413) (Table 3). We used the threshold of age> =60 in the multivariable logistic regression model because of the dichotomized distribution of the age of deceased patients (Supplementary Figure 1): out of the 52 deceased patients, 40 were 60 years old or older. This pattern is consistent with the reports that age >60 was a risk factor for critical illness in COVID-19 (17, 18). Similar result was obtained with survival analysis: after adjusting for the effects of age and other confounding factors, mutivariable analysis with the Cox proportional-hazards model indicated a lower survival rate in the CS group (HR 4.52 [95%CI: 1.79–11.41], *p* = 0.001, Supplementary Table 1). In line with higher case fatality rate, more of the patients treated with corticosteroids presented ARDS (CS vs. non-CS: 63 vs. 36%) and exacerbation of pulmonary fibrosis (CS vs. non-CS: 56 vs. 21%) than patients not treated with corticosteroids (Table 2). A higher proportion of the CS group required mechanical ventilation than the non-CS group, but statistical significance was not achieved. Among the surviving patients, the length of hospital stay was higher in the CS group [24 (IQR: 18.3–38.8) days] than in the non-CS group [17 (13.8–25.3) days]. The length of virus clearance time was also longer in the CS group [21 (16.8–26.3) days] than in the non-CS group [13.5 (12–16) days].

**Table 2:** Outcomes of critical COVID-19 patients on admission.

**Outcomes**	**Corticosteroid treatment (*n* = 68)**	**Non-corticosteroid treatment (*n* = 28)**	***P*-value**
ARDS	43 (63%)	10 (36%)	**0.022**
Mechanical ventilation	36 (53%)	10 (36%)	0.143
ICU admission	45 (66%)	21 (75%)	0.397
Case fatality	46 (68%)	6 (21%)	**0.001**
Exacerbation of pulmonary fibrosis	38 (56%)	6 (21%)	**0.005**
SOFA	10.50 (5.25–13.00)	5.00 (4.00–7.00)	**0.006**
Length of hospital stay (survival), days	24.0 (18.3–38.8)	17.0 (13.8–25.3)	**0.041**
Length of virus clearance time (survival), days	21.0 (16.8–26.3)	13.5 (12.0–16.0)	**0.004**

*Data are median (IQR) or n (%). P-values comparing corticosteroid treatment and non-corticosteroid treatment are from Mann-Whitney U test, Chi-Square test, or Fisher's exact test, as appropriate, and adjusted for multiple comparisons by FDR method. ARDS, acute respiratory distress syndrome; ICU, intensive care unit; SOFA, sequential organ failure assessment. The bold P-values indicate P < 0.05*.

**Table 3:** Logistic univariate and multivariate regression analyses on the association between the case fatality rate and the clinical features.

	**Univariable OR (95% CI)**	***P*-value**	**Multivariable OR (95%CI)**	***P*-value**
Age ≥ 60years	2.10 (0.87–5.09)	0.101	0.73 (0.21–2.58)	0.630
Sex	0.79 (0.35–1.80)	0.580	0.82 (0.29–2.36)	0.711
**Comorbidities**				
Hypertension	2.44 (1.06–5.59)	**0.035**	3.29 (1.08–10.00)	**0.036**
Diabetes	1.51 (0.50–4.54)	0.466		
**Treatments**			
Antiviral	1.21 (0.33–4.47)	0.780		
Interferon	8.51 (1.83–39.72)	**0.006**	2.67 (0.37–19.15)	0.328
Antibiotics	0.98 (0.28–3.47)	0.979		
Corticosteroids	7.67 (2.72–21.60)	**< 0.001**	4.05 (1.37–11.98)	**0.012**
Intravenous immunoglobulin	6.64 (1.80–24.54)	**0.005**	3.54 (0.54–23.13)	0.187
Albumin	1.29 (0.21–8.06)	0.788		
Thymosin α1	0.09 (0.01–0.74)	**0.025**	0.025 (0.00–0.36)	**0.007**

*OR, odds ratio; CI, confidence interval; ARDS, acute respiratory distress syndrome; SOFA, sequential organ failure assessment. Multivariable logistic regression were adjusted for age, sex, hypertension, interferon, corticosteroids, intravenous immunoglobulin, and thymosin α1. The bold P-values indicate P < 0.05*.

### Laboratory Findings

For better understanding of the elevated case fatality in the CS group, we reviewed the laboratory findings for blood cell counts, markers for coagulation function, inflammatory biomarkers and the markers for cellular, tissue and organ damage.

On admission, no difference in laboratory findings was observed between the CS and the non-CS groups (Table 4). Decreased lymphocyte counts in both groups of critical patients reflected the inflammatory characteristics of these patients. Other outstanding observations included the elevated CRP, ESR, and LDH in both groups (Table 4), reflecting viral infections, inflammatory reactions and tissue damage.

**Table 4:** Laboratory findings of critical COVID-19 patients on admission.

	**Reference values**	**Corticosteroid treatment**	**Non-corticosteroid treatment**	***P*-value**
		**(*n* = 68)**	**(*n* = 28)**	
White blood cell count (×10^9^/L)	4.00–10.00	7.79 (5.12–10.95)	7.94 (5.29–11.93)	0.763
Lymphocyte count (×10^9^/L)	1.50–4.00	0.81 (0.61–1.13)	1.01 (0.75–1.59)	0.551
Neutrophil count (×10^9^/L)	2.00–7.00	6.25 (3.55–8.88)	5.72 (3.31–8.99)	0.856
NLR	0.78–3.53	7.05 (4.26–11.71)	3.93 (2.22–9.87)	0.856
Monocyte count (×10^9^/L)	0.12–1.00	0.35 (0.21–0.53)	0.45 (0.26–0.73)	0.551
Platelet count (×10^9^/L)	99.00–303.00	187.00 (157.75–257.00)	244.00 (167.75–285.75)	0.763
APTT(s)	21.00–37.00	29.13 (24.33–35.63)	34.64 (27.50–37.45)	0.551
fibrinogen (g/L)	2.00–4.00	3.46 (2.76–4.47)	3.38 (2.32–4.84)	0.856
D-dimer (μg/ml)	0.00–0.55	0.57 (0.36–1.15)	0.55 (0.34–1.26)	0.856
Procalcitonin (ng/ml)	0.00–0.50	0.26 (0.15–0.44)	0.35 (0.23–0.42)	0.881
CRP(mg/L)	0.00–10.00	24.45 (9.78–100.19)	27.70 (8.97–70.47)	0.763
ESR (mm/1 h)	0.00–30.00	36.00 (26.15–66.50)	32.00 (26.00–49.00)	0.763
Creatine kinase (U/L)	25.00–200.00	150.00 (64.00–175.50)	166.50 (85.90–408.75)	0.551
Lactate dehydrogenase (U/L)	91.00–230.00	230.00 (171.15–350.40)	234.40 (191.00–323.50)	0.856
Creatinine (μmol/L)	44.00–112.00	82.50 (67.50–107.00)	78.70 (61.90–110.40)	0.763
BUN (mmol/L)	2.50–7.10	6.57 (4.80–11.03)	6.52 (5.41–7.50)	0.856
AST (U/L)	0.00–40.00	37.35 (27.00–55.75)	50.45 (32.75–66.50)	0.856
ALT (U/L)	0.00–50.00	42.50 (28.00–63.60)	37.00 (29.25–55.53)	0.763
Total bilirubin (μmol/L)	3.00–21.00	20.55 (14.10–28.65)	17.85 (14.97–24.98)	0.763

*Data are median (IQR). P-values comparing corticosteroid treatment and non-corticosteroid treatment are from Mann-Whitney U test or Student's t test, as appropriate, and adjusted for multiple comparisons by FDR method. NLR, neutrophil-to-lymphocyte ratio; APTT, activated partial thromboplastin time; ESR, erythrocyte sedimentation rate; CRP, C-reactive protein; BUN, blood urea nitrogen; AST, aspartate transaminase; ALT, alanine aminotransferase*.

After corticosteroid treatments, patients treated with corticosteroids were associated with higher counts of white blood cells, neutrophils, and neutrophil-to-lymphocyte ratio (Table 5). The differences in the counts of white blood cells remained significant after adjustment for confounding factors (Table 6).

**Table 5:** Laboratory findings of critical COVID-19 patients before discharge.

	**Reference values**	**Corticosteroid treatment**	**Non-corticosteroid treatment**	***P*-value**
		**(*n* = 68)**	**(*n* = 28)**	
White blood cell count (×10^9^/L)	4.00–10.00	8.48 (5.88–12.78)	6.90 (4.32–8.46)	**0.015**
Lymphocyte count (×10^9^/L)	1.50–4.00	0.75 (0.54–1.12)	0.86 (0.52–1.33)	0.475
Neutrophil count (×10^9^/L)	2.00–7.00	6.44 (3.90–10.83)	5.19 (2.75–7.29)	**0.015**
NLR	0.78–3.53	9.57 (4.70–16.21)	6.12 (2.43–9.67)	**0.015**
Monocyte count (×10^9^/L)	0.12–1.00	0.45 (0.28–0.65)	0.48 (0.25–0.66)	0.692
Platelet count (×10^9^/L)	99.00–303.00	185.00 (125.75–249.75)	233.00 (128.50–300.50)	0.100
APTT(s)	21.00–37.00	33.00 (25.75–41.00)	35.50 (32.10–49.25)	0.440
fibrinogen (g/L)	2.00–4.00	3.26 (2.03–4.23)	3.50 (2.13–4.48)	0.692
D-dimer (μg/ml)	0.00–0.55	1.05 (0.48–6.66)	0.54 (0.34–2.52)	0.642
Procalcitonin (ng/ml)	0.00–0.50	0.32 (0.12–0.58)	0.34 (0.21–0.44)	0.692
CRP(mg/L)	0.00–10.00	61.70 (24.88–156.85)	42.41 (15.25–122.63)	0.475
ESR (mm/1 h)	0.00–30.00	46.00 (32.00–78.50)	46.00 (31.25–81.75)	0.719
Creatine kinase (U/L)	25.00–200.00	178.00 (50.93–357.25)	187.50 (98.80–384.00)	0.903
Lactate dehydrogenase (U/L)	91.00–230.00	314.00 (214.00–579.00)	309.00 (210.00–393.00)	0.692
Creatinine (μmol/L)	44.00–112.00	83.40 (63.90–145.25)	84.50 (66.13–129.75)	0.692
BUN (mmol/L)	2.50–7.10	7.80 (5.33–18.85)	7.60 (4.99–14.47)	0.692
AST (U/L)	0.00–40.00	64.00 (41.00–112.00)	62.00 (46.75–78.00)	0.692
ALT (U/L)	0.00–50.00	67.00 (46.00–133.00)	61.00 (41.25–114.00)	0.692
Total bilirubin (μmol/L)	3.00–21.00	28.70 (19.65–34.13)	22.15 (17.98–30.80)	0.692
SOFA		10.50 (5.25–13.00)	5.00 (4.00–7.00)	**0.015**

*Data are median (IQR). P-values comparing corticosteroid treatment and non-corticosteroid treatment are from Mann-Whitney U test or Student's t test, as appropriate, and adjusted for multiple comparisons by FDR method. NLR, neutrophil-to-lymphocyte ratio; APTT, activated partial thromboplastin time; ESR, erythrocyte sedimentation rate; CRP, C-reactive protein; BUN, blood urea nitrogen; AST, aspartate transaminase; ALT, alanine aminotransferase; SOFA, sequential organ failure assessment. The bold P-values indicate P < 0.05*.

**Table 6:** Logistic univariate and multivariate regression analyses on the association between the corticosteroid treatment and the clinical features.

	**Univariable OR (95% CI)**	***P*-value**	**Multivariable OR (95% CI)**	***P*-value**
**Age≥60 years**	3.53 (1.39–9.02)	**0.008**	4.94 (1.49–16.41)	**0.009**
**Sex**	0.70 (0.28–1.75)	0.449		
**Laboratory findings**				
White blood cell count (X10^9^/L)	1.15 (1.02-1.30)	**0.022**	1.19 (1.03-1.37)	**0.019**
4–10	ref			
<4	0.38 (0.10–1.39)	0.142		
>10	2.59 (0.78–8.58)	0.120		
Lymphocyte count (×10^9^/L)	0.58 (0.31–1.08)	0.085		
1.5–4.0	ref		ref	
<1.5	2.61 (1.61–4.22)	**<0.001**	1.79 (0.63–5.06)	0.271
>4.0^#^	/	/		
Neutrophil count (×10^9^/L)	1.16 (1.02–1.31)	**0.023**	1.21 (1.04–1.40)	**0.016**
2–7	ref		ref	
<2	1.00 (0.25–4.00)	1.000		
>7	4.43 (1.95–10.06)	**<0.001**	2.32 (0.82–6.61)	0.115
NLR	1.13 (1.03–1.23)	**0.008**	1.16 (1.03–1.29)	**0.011**
0.78–3.53	ref		ref	
<0.78^#^	/	/		
>3.53	3.22 (1.90–5.47)	**<0.001**	3.51 (1.30–9.49)	**0.013**
Monocyte count (×10^9^/L)	1.58 (0.34–7.46)	0.561		
0.12–1.00	ref			
<0.12^#^	/	/		
>1.00^#^	/	/		
Platelet count (×10^9^/L)	0.99 (0.99–1.00)	**0.029**	0.99 (0.99–1.00)	0.059
99–303	ref			
<99	2.75 (0.88–8.64)	0.083		
>303	0.80 (0.22–2.98)	0.739		
APTT(s)	0.97 (0.94–1.01)	0.136		
21–37	ref			
<21	1.00 (0.63–15.99)	1.000		
>37	1.77 (0.90–3.49)	0.100		
Fibrinogen (g/L)	0.93 (0.70–1.23)	0.596		
2–4	ref			
<2	3.00 (1.09–8.25)	**0.033**	1.40 (0.37–5.34)	0.626
>4	1.55 (0.72–3.30)	0.261		
D-dimer (μg/mL)	1.05 (0.96–1.15)	0.293		
0-0.55	ref		ref	
>0.55	3.14 (1.72–5.74)	**<0.001**	2.67 (1.00–7.15)	0.050
Procalcitonin (ng/mL)	1.22 (0.74–2.03)	0.440		
0-0.50	ref		ref	
>0.50	4.25 (1.43–12.63)	**0.009**	2.27 (0.62–8.40)	0.218
CRP(mg/L)	1.00 (1.00–1.01)	0.193		
0–10	ref		ref	
>10	2.61 (1.61–4.22)	**<0.001**	1.63 (0.59–4.44)	0.344
ESR (mm/1h)	1.00 (0.99–1.01)	0.679		
0-30	ref		ref	
>30	2.05 (1.23–3.41)	**0.006**	1.29 (0.48–3.53)	0.614
Creatine kinase (U/L)	1.00 (1.00–1.00)	0.902		
25–200	ref		ref	
<25	2.00 (0.18–22.06)	0.571		
>200	2.75 (1.22–6.18)	**0.014**	1.30 (0.40–4.28)	0.663
Lactate dehydrogenase (U/L)	1.00 (1.00–1.00)	0.546		
44–112	ref		ref	
<44^#^	/	/		
>112	2.06 (1.15–3.68)	**0.015**	1.01 (0.38–2.72)	0.981
Creatinine (μmol/L)	1.00 (1.00–1.01)	0.588		
91–230	ref		ref	
<91^#^	/	/		
>230	3.12 (1.41–6.93)	**0.005**	2.10 (0.71–6.20)	0.180
BUN (mmol/L)	1.00 (0.99–1.01)	0.627		
2.5–7.1	ref		ref	
<2.5	2.00 (0.18–22.06)	0.571		
>7.1	2.40 (1.31–4.38)	**0.004**	1.38 (0.56–3.42)	0.485
AST (U/L)	1.00 (1.00–1.00)	0.620		
0–40	ref		ref	
>40	2.22 (1.36–3.63)	**0.002**	0.89 (0.32–2.46)	0.817
ALT (U/L)	1.00 (1.00–1.00)	0.620		
0–50	ref		ref	
>50	3.13 (1.75–5.60)	**<0.001**	2.90 (1.06–7.97)	**0.039**
Total bilirubin (μmol/L)	1.01 (0.97–1.05)	0.544		
0–21	ref		ref	
>21	3.29 (1.81–5.98)	**<0.001**	2.36 (0.85–6.55)	0.098
SOFA	1.17 (1.05–1.31)	**0.005**	1.21 (1.07–1.38)	**0.004**
1	ref		ref	
2–5	1.21 (0.60–2.46)	0.591	1.11 (0.31–3.95)	0.872
6–10	2.83 (1.12–7.19)	**0.028**	1.82 (0.38–8.64)	0.453
>10	5.67 (2.34–13.50)	**<0.001**	6.31 (1.34–29.73)	**0.020**

*OR, odds ratio; CI, confidence interval. Multivariable logistic regression for age were adjusted for sex and comorbidities. Multivariable logistic regression for laboratory findings were adjusted for age, sex and comorbidities. NLR, neutrophil-to-lymphocyte ratio; APTT, activated partial thromboplastin time; ESR, erythrocyte sedimentation rate; CRP, C-reactive protein; BUN, blood urea nitrogen; AST, aspartate transaminase; ALT, alanine aminotransferase; SOFA, sequential organ failure assessment. The bold P-values indicate P < 0.05*.

# *Too few cases*.

No difference was observed for coagulation function markers APTT, fibrinogen or d-dimer (Table 5). Serum markers for inflammation CRP and ESR were elevated in both the CS and the non-CS groups, but not different between the study groups.

Regarding markers for tissue and organ damage, multivariate logistic regression revealed higher incidence of elevated ALT and SOFA score > 10 in the CS group (Table 6). Levels of LDH, BUN, AST, and total bilirubin were elevated in both the CS and the non-CS group, but not different between the groups (Table 5).

## Discussion

Our data showed that critical COVID-19 patients treated with corticosteroid had a much higher case fatality rate than those not treated with corticosteroid. This is in line with our observations that increased incidences of ARDS and exacerbation of pulmonary fibrosis, longer hospital stay and virus clearance time were associated with the use of corticosteroid in critical COVID-19 patients. Further, laboratory results provided evidence for increased incidence of tissue damage in corticosteroid users.

One immediate question is which came first, more severe symptoms or the use of corticosteroids? Use of corticosteroids was likely the lead because patients in the two study groups were both critically ill, with similar symptoms and laboratory results on admission. One possible argument is that patients in the CS group were perceived sicker by the physicians, because these patients were significantly older, and many patients in the CS group were treated with interferon, immunoglobulin and albumin, which were not prescribed for the patients in the non-CS group. To address this concern, the influences of age, possible more severe symptoms represented by the use of interferon and immunoglobulin, as well as other confounding factors, were adjusted in the multivariate logistic regression, resulted in an OR of 4.05 (1.37–11.98) for case fatality, indicating that corticosteroid use caused a much higher case fatality in critical patients.

In our hospitals, corticosteroids were usually used in the second week of disease onset when severe symptoms presented. In theory, this is a good timing for the suppression of inflammatory reactions that cause damage in ARDS and viral pneumonia (19). However, the beneficial outcomes seen with SARS patients and other types of pneumonia did not occur with our COVID-19 patients. Instead, we observed higher case fatality rate and other adverse events in critical patients treated with corticosteroids. Although pulmonary dysfunction was common in our patients treated with corticosteroids, elevated SOFA scores of these patients indicated that multi-organ failure often contributed to corticosteroid related death. In support of this, blood biochemistry revealed frequent incidence of liver injury in corticosteroid treated patients. The multi-organ failure was likely caused by more persistent viral infection and higher viral load, which could be a consequence of the inhibition of the body's immune surveillance in corticosteroid treated patients. Our retrospective study is very limited on relevant data for the evaluation of the corticosteroid effects on patient immunity and subsequent infection risk (viral load, cytokine levels, etc). Available data related to immunity and subsequent infection risk include blood cell counts, inflammation markers and viral clearance time. Before discharge, white blood cell count, lymphocyte count, and NLR were higher in the corticosteroid group, a reflection of being recovered from viral infection. As it is well-established that corticosteroid reduces inflammatory activities in viral pneumonia (19), our data support that, patients' immunity was suppressed by short-term corticosteroid treatment, which may lead to increased viral replication, extended viral clearance, and secondary infections, and therefore subsequent inflammatory reactions in multiple organs. In addition, corticosteroid treatment may directly affect SARS-CoV-2 replication, similarly as it increases viral replication of rhinovirus and influenza A virus through reduced expression of innate anti-viral genes (20). Although data is not available in our patients, known adverse effects of corticosteroids include pancreatitis (21), heart failure, ischemic heart disease (22), and myopathy (23), which may contribute to the elevated SOFA score in corticosteroid treated patients.

Our observations are different from some of the published studies. Zha et al. examined the outcome of 11 mild COVID-19 patients treated with corticosteroid (24). Compared with patients not treated with corticosteroid, the treated patients exhibited no significant difference in every outcome measures, likely due to the small sample size. However, it is noteworthy that the corticosteroid treated patients consistently exhibited a larger median for the virus clearance time, for the length of hospital stay, and for the duration of symptoms.

Recently, in contrast to our findings, two randomized open-label trials reported beneficial effects of corticosteroid therapy on severe COVID-19. The RECOVERY study reported reduced case fatality in patients treated with corticosteroid (25), while the similarly designed REMAP-CAP study observed an increased odds of improvement in organ support-free days within 21 days in the corticosteroid treatment group (26). Inconsistently, data from the REMAP-CAP study also indicated a trend of increased length of ICU stay and hospital stay for the corticosteroid treated patients. One of the limitations in both REMAP-CAP and RECOVERY studies, as reported by the authors, was that 15% of the no corticosteroid group received systemic corticosteroids. Another important limitation in both studies was that they enrolled suspected COVID-19 patients. Without positive viral RNA diagnosis, these studies provide little support for beneficial effects of corticosteroid therapy in COVID-19.

It is worth noting that one earlier review summarizing all data on corticosteroid use with COVID-19 before July 2020 concluded that corticosteroid therapy is associated with the improvement in symptoms and oxygenation for individuals with severe COVID-19, but case fatality rate in the corticosteroid group was significantly higher than that in the non-corticosteroid group (27). This conflict between improved symptoms and increased case fatality rate could be a consequence of the heterogeneous nature of the different studies being reviewed. In contrast, we observed, consistently, higher case fatality rate and higher odds for other adverse outcomes after corticosteroid treatment, which may due to the strict enforcement of our inclusion criteria.

Conflicting results have also been reported on the use of corticosteroids in ARDS and infectious pneumonia. Studies from Meduri' group (28–31), including a randomized, double-blind, placebo-controlled trial (28), and other groups (32–35) reported beneficial effects of methylprednisolone on ARDS. However, in a prospective, randomized, double-blind, placebo-controlled trial in 99 patients with ARDS, high dose of methylprednisolone did not affect case fatality, the reversal of ARDS, or the incidence of secondary bacterial infection (36). Similarly, in a prospective, randomized, double-blind, placebo-controlled trial of treating severe sepsis and septic shock with high-dose methylprednisolone, no significant difference were found in the prevention or reversal of shock, or overall case fatality of the patients (37). Adverse outcomes from corticosteroid treatment in sepsis were also frequently reported and summarized in a meta-analysis, which concluded that corticosteroid treatment increased case fatality rate and caused a trend of increased rate of secondary infection (38).

In a retrospective cohort study similarly designed as our study, Auyeung et al. examined the effect of corticosteroid in the treatment of SARS patients (39). They reported a 20.7-fold increase in risk of either ICU admission or case fatality, after adjusting for the effects of age and disease severity. Extrapulmonary injury may have contributed to the adverse outcomes, as their corticosteroid treated patients exhibited a trend of elevated lactate dehydrogenase. These results suggest that, although damage caused by inflammation in SARS and COVID-19 pneumonia can be lethal, turning off the immune surveillance with corticosteroids may cause additional adverse outcomes. Both SARS-CoV-2 and SARS-CoV are known to infect lung, intestines and other organs (40, 41), and the use of corticosteroid may exacerbate the viral infections leading to multi-organ failure.

Other limitations of our study include small sample size and imbalanced age between groups. The sample size did not allow sufficient power for many of the comparisons between the study groups. We are lucky that, an *R*^2^ value of 0.413 was achieved in the multivariable logistic regression of the case fatality rate, indicating a decent power for the analysis of the primary outcome. The age of the CS group was apparently older and the difference was statistically significant before adjustment for multiple comparisons. Considering the potential impact of the age on the clinical outcomes, age was included in the multivariate regressions. Our results indicated that adverse outcomes remained significantly associated with corticosteroid use in critical COVID-19 after adjusting for the influence of age and other confounding factors.

In conclusion, corticosteroid use in critically ill COVID-19 patients was associated with a much higher case fatality rate. Frequent incidence of liver injury and multi-organ failure in corticosteroid treated patients may have contributed to the adverse outcomes. The multi-organ failure is likely caused by more persistent SARS-CoV-2 infection and higher viral load, due to the inhibition of immune surveillance by corticosteroid.

## Data Availability Statement

The raw data supporting the conclusions of this article will be made available by the authors, without undue reservation.

## Ethics Statement

The studies involving human participants were reviewed and approved by The Institutional Review Board of the Six Affiliated Hospital of Sun Yat-sen University. Written informed consent for participation was not required for this study in accordance with the national legislation and the institutional requirements.

## Author Contributions

GY, LZ, and RZ conceived and designed this study. YL, JL, YZ, LZ, SC, and YH collected data. YL, JK, NJ, JL, LZ, GY, and RZ analyzed data. YL, JK, NJ, and LZ prepared the first draft. All authors critically revised the manuscript and approved the final version.

## Conflict of Interest

The authors declare that the research was conducted in the absence of any commercial or financial relationships that could be construed as a potential conflict of interest.
