# Supplementary figures and images for: Adverse Outcomes Associated With Corticosteroid Use in Critical COVID-19: A Retrospective Multicenter Cohort Study
Source: Front Med (Lausanne). 2021 Feb 10;8:604263. doi: 10.3389/fmed.2021.604263 (PMC7900536; doi:10.3389/fmed.2021.604263)

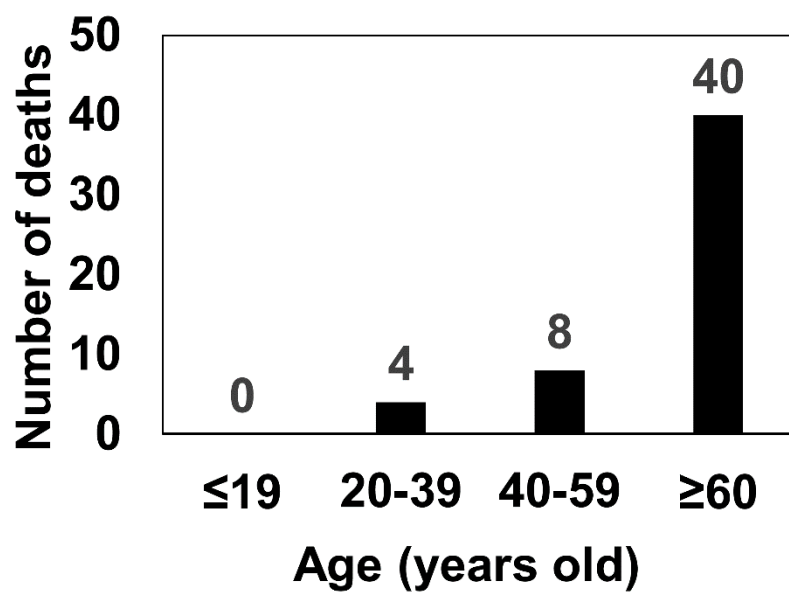

Supplementary Figure 1, Age distribution of the deceased COVID-19 patients.

Supplement: Supplementary file 1 [file Image_1.pdf]
