# Supplementary material for: Adverse Outcomes Associated With Corticosteroid Use in Critical COVID-19: A Retrospective Multicenter Cohort Study
Source: Front Med (Lausanne). 2021 Feb 10;8:604263. doi: 10.3389/fmed.2021.604263 (PMC7900536; doi:10.3389/fmed.2021.604263)
Supplement: Supplementary file 2 [file Table_1.docx]

| Supplementary Table 1, The effects of corticosteroid use on survival: the multivariable Cox proportional-hazards model | | | |
| --- | --- | --- | --- |
|  | Multivariable HR (95%CI) | *P*-value | |
| Age≥60years | 0.78 (0.37-1.66) | | 0.515 |
| Sex | 1.11 (0.63-1.99) | | 0.715 |
| Hypertension | 2.15 (1.17-3.94) | | **0.014** |
| Interferon | 1.17 (0.53-2.55) | | 0.702 |
| Corticosteroids | 4.52 (1.79-11.41) | | **0.001** |
| Intravenous immunoglobulin | 1.25 (0.59-2.67) | | 0.560 |
| Thymosin α1 | 0.11 (0.02-0.79) | | **0.028** |
|  |  | |  |
| Multivariable Cox proportional-hazards analysis were adjusted for age, sex, hypertension, interferon, corticosteroids, intravenous immunoglobulin, and thymosin α1. The bold P-value indicate P＜0.05. | | | |
